# Factors influencing glycocalyx degradation: a narrative review

**DOI:** 10.3389/fimmu.2024.1490395

**Published:** 2025-01-16

**Authors:** Jing Wang, Lan Ma, Yu Fang, Tengteng Ye, Hongbo Li, Peng Lan

**Affiliations:** ^1^ Department of Cardiology, Nanning Hospital of Traditional Chinese Medicine, Nanning, Guangxi, China; ^2^ Department of Neurology, Wenzhou Traditional Chinese Medicine (TCM) Hospital of Zhejiang Chinese Medical University, Wenzhou, Zhejiang, China

**Keywords:** cell membrane structures, endothelium, inflammation, review, glycocalyx

## Abstract

The glycocalyx is a layer of villus-like structure covering the luminal surface of vascular endothelial cells. Damage to the glycocalyx has been proven linked to the development of many diseases. However, the factors that promote damage to the glycocalyx are not fully elaborated. This review summarizes factors leading to the reduction of the glycocalyx in detail, including inflammatory factors, ischemia-reperfusion, oxidative stress, lipids, glucose, high sodium, female sex hormones and others. Additionally, the mechanisms underlying its degradation are discussed. To better prevent and treat related diseases induced by glycocalyx degradation, it is a meaningful measure to avoid these factors.

## 1 Introduction

In 1940, Danielli (1) first discovered a thin layer adhering to the surface of vascular endothelium and distinguished it from the plasma proteins of the cell, providing indirect evidence for the discovery of the glycocalyx. In 1956, Springer (2) identified polysaccharides as the primary components of the non-cellular outer layer of the endothelium, laying the theoretical foundation for the richness of polysaccharides in the glycocalyx. In 1963, Bennett (3) coined “glycocalyx” to refer to the polysaccharide-rich endothelial coat. In 1966, Luft (4) directly observed the biological structure of the glycocalyx using ruthenium red staining and microscopic examination, providing direct evidence of the structural characteristics of the glycocalyx. It could be seen that glycocalyx’s structural complexity and detection challenges have slowed research progress in the past few decades. With the rapid development of detection technologies, various new detection and analysis techniques have emerged, accelerating the pace of glycocalyx research (5). Based on studies of glycocalyx structure, an increasing number of researchers are exploring its functions and discovering its critical role in protecting vascular endothelium. They also uncover its biological relevance to the occurrence and development of multiple diseases. Therefore, as a therapy pathway, protecting the glycocalyx to maintain the endothelial barrier function has recently become a research hotspot in disease prevention and treatment.

Numerous prior studies have summarized the physiology and pathology of the glycocalyx (6, 7), such as Bernhard F. Becke’s examination of various clinical conditions influencing glycocalyx damage, the enzymes responsible for its shearing, and potential protective strategies (8). To the best of our knowledge, a detailed discussion on the mechanisms underlying glycocalyx damage remains absent. Sietze Reitsma primarily focused on the composition, structure, and function of the glycocalyx (9), whereas S. Tumova concentrated on the biological roles of heparan sulfate (10). These papers predominantly concentrate on the function of the glycocalyx. Thus, it is indispensable to write an exhaustive review that explores both the factors contributing to and the mechanisms underlying glycocalyx damage. Understanding these inducing mechanisms is crucial for disease prevention and treatment, thereby enabling more targeted intervention strategies. This review aims to elucidate the factors and mechanisms associated with glycocalyx damage to provide valuable insights for preventing and treating related diseases.

## 2 Structure and synthesis of the glycocalyx

**Figure 1:**
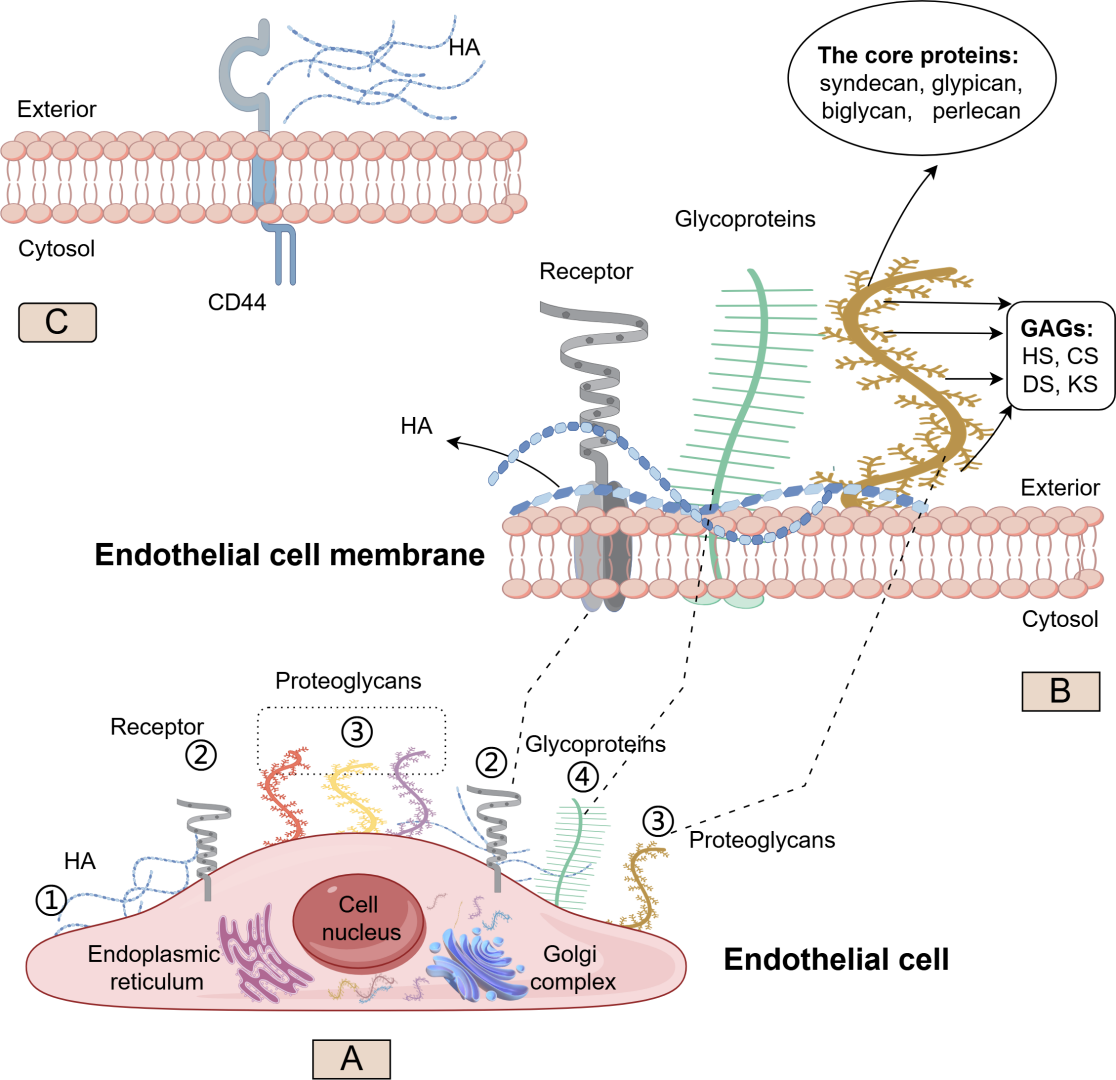
Structure and synthesis of the glycocalyx. Schematic representation of the major glycocalyx components covering the luminal surface of endothelial cells (EC). On **(A)**, ① Hyaluronan (HA) (blue) synthesized on the cellular surface; ② Cell surface receptor (gray); ③ Four examples of proteoglycans (PGs) (red, yellow, purple, brown) carry long glycosaminoglycan (GAG) side chains; ④ Other glycoproteins (green) have shorter, unbranched carbohydrate side chains. On **(B)**, the structure of HA (blue), other glycoproteins (green) and PGs (brown) on phospholipid bilayer cell membrane; the core protein (including syndecan, glypican, biglycan, and perlecan) and GAG sidechain (including heparan sulfate (HS), chondroitin sulfate (CS), keratan sulfate (KS), and dermatan sulfate (DS)) in PGs On **(C)**, the interaction of HA with the CD44 glycoprotein. (By Figdraw).

The glycocalyx is a negatively charged, villus-like structure that covers the surface of vascular endothelium, which extends from a few nanometers to several micrometers and much longer than endothelial cell receptors (11, 12). It serves as a natural barrier between the endothelium and the blood. The glycocalyx is composed of various proteoglycans (PG) and glycoproteins (**Figure 1**). Glycoproteins link to oligosaccharide chains containing terminal sialic acid residues. PGs consist of membrane-anchored core proteins covalently linked to negatively charged glycosaminoglycan (GAG) side chains through a tetrasaccharide. The physicochemical properties of the GAG chains depend on their specific polysaccharide components. Typical components of GAG include heparan sulfate (HS), chondroitin sulfate (CS), keratan sulfate (KS), dermatan sulfate (DS), and hyaluronan (HA) (9), in which HS is the most abundant, accounting for 50%∼90% of the total GAG (13). Most of these components have high sulfate characteristics, making the glycocalyx a negatively charged structure that protects the endothelium. The core proteins anchored to the endothelial cell membrane mainly include syndecan, glypican, biglycan, and perlecan. These molecules can combine with the five types mentioned above of GAGs to create different types of glycocalyx, playing a complex and diverse role in the interaction between vascular endothelium and plasma. However, it is important to note that HA is a free polysaccharide not covalently attached to a core protein and lacks sulfate groups in its structure. It obtains its negative charge from carboxyl groups that endow it with exceptional hydration properties. In brief, the glycocalyx structure composed of different components is essential for maintaining the integrity and physiological function of endothelial cells.

Aside from HA, which is synthesized directly on the cell surface, the core proteins and polysaccharide components of PGs are primarily synthesized in the endoplasmic reticulum and Golgi apparatus. Once modified by various GAGs, PGs can be transported from the Golgi to the cell surface, embedded in the membrane, and exert physiological functions.

## 3 Physiology and pathology of the glycocalyx

The primary physiological function of the glycocalyx is to protect the endothelium. It plays a crucial role in maintaining normal vascular function by serving the glycocalyx as a bridge between circulating substances and endothelial cells, acting as a selective permeability barrier of the vessel wall, and functioning as a mechanical sensor of blood shear forces (14). Therefore, the glycocalyx is essential for maintaining vascular homeostasis by preventing the adhesion of blood cells to the endothelium, regulating vascular permeability, and modulating the transduction of mechanical signals from blood flow (15).

Syndecans, as quintessential core proteins, not only interact with GAGs to fulfill their functional roles but also serve as co-receptors for various growth factors (16). They play a pivotal role in modulating growth factor signaling pathways and exhibit distinct physiological functions. For instance, when fibroblast growth factor (FGF) binds to its receptor on the cell surface, syndecans can enhance the affinity of FGF for its receptor, facilitate the dimerization and activation of the FGF receptor, thereby amplifying FGF signal transduction (17). Moreover, during the assembly and remodeling of the extracellular matrix, syndecans play a crucial role in regulating both the deposition and organization of extracellular matrix components, which in turn influences their physical properties and biological functions. In specific contexts, such as tissue damage repair, syndecans are instrumental in regulating collagen synthesis and deposition, thereby promoting wound healing and tissue remodeling (18).

The GAG side chain HS has binding sites for various growth factors, cytokines, chemokines, and enzymes, including extracellular superoxide dismutase, endothelial nitric oxide synthase (eNOS), angiotensin-converting enzyme, lipoprotein lipase, antithrombin-III (10). Substances in circulation can bind to sites on the glycocalyx, increasing its stability, preventing glycocalyx degradation, maintaining specific concentrations on the cell surface, promoting intracellular signal transduction, and inhibiting coagulation and inflammatory reactions within vessels. The surface-bound glycocalyx GAG constituent HS is crucial for complement factor H binding and function, both in the recognition of host tissue and prevention of spontaneous complement activation via the alternative pathway (19). HS also plays an important role in the recruitment, rolling and firm adhesion of leucocytes to activated endothelium. Normal HS structure can reduce the number of white blood cell adhesions and reduce the degree of inflammation (20, 21). For HA, it can bind to the transmembrane glycoprotein CD44 on the cell surface (22), exhibiting high affinity and specificity (**Figure 1C**). CD44 localizes along with HA in caveolae, where it has various functional interactions. A single CD44 molecule can interact with multiple HA molecules, resulting in the formation of a CD44-HA complex that mediates adhesion between cells and the extracellular matrix. This binding mechanism is crucial for how cells sense and respond to alterations in the extracellular matrix microenvironment (23). If the glycocalyx degrades and sheds, its interaction with proteins, cells, and other substances in the circulation disappears, creating conditions for coagulation and inflammation within vessels. Research has indicated endothelial loss of HA results in disturbed glomerular endothelial stabilization (24).

Many important receptors on the cell surface, including selectins, integrins, and vascular endothelial cadherin, possess oligosaccharides and are classified as glycoproteins (25). Under normal physiological conditions, P-selectin in endothelial cells is typically stored in intracellular secretory particles, such as Weibel-Palade bodies. When the cell is stimulated and activated, this stored P-selectin is rapidly transported to the cell surface, resulting in a significant increase in its expression, thereby facilitating its biological functions (26). Integrins, which are glycosylated glycoproteins, can bind to fibronectin and collagen in the extracellular matrix, enhancing adhesion between cells and the extracellular matrix. This interaction provides a stable environment for cell growth and survival, while also participating in processes such as cell migration and proliferation (27). Tight junctions are essential for maintaining vascular permeability as barriers. For example, cadherin, a glycoprotein, mediates homophilic adhesion between cells. It interacts with cadherin on the surfaces of adjacent cells through glycosylation sites located in the extracellular domains, thereby establishing adhesion connections such as tight junctions, desmosomes, and adhesion bands, which are vital for maintaining the stability of tissues (28).

Because of its charge selectivity, the glycocalyx can prevent negatively charged substances from entering endothelial cells or passing through intercellular gaps, thereby preserving the integrity of the blood vessel walls (29). Researchers observed that anions migrate to the luminal side of the glycocalyx at a slower rate than neutral dextran, demonstrating the involvement of the glycocalyx’s negative charge in maintaining vascular permeability (30). The maintenance of vascular tone is closely linked to nitric oxide (NO) production. Vascular shear stress, a crucial regulator of NO production, can transmit signals to cells by interacting with the glycocalyx, induces the expression of eNOS, and enhances NO generation in endothelial cells (31, 32). Studies reversely confirmed that damage to the glycocalyx inhibited the expression of eNOS and NO production and affected flow-mediated vasodilation (33–35). This effect was comparable to using eNOS inhibitors, indicating that an intact glycocalyx is necessary for maintaining eNOS function (36). Moreover, syndecans could transmit sensed blood flow shear stress signals into cells through interactions between their extracellular domains and intracellular connexin proteins, promoting endothelial cells to align along the direction of blood. The alignment maintained the normal function of tight junction complexes and the normal flow state of blood (37). Both *in vivo* and *in vitro* experiments confirmed this finding: the organized cellular arrangement in the direction of blood flow was disrupted when syndecan-4 was knocked out (38).

## 4 Methods for the detection of glycocalyx

It is important to note that employing appropriate detection methods to observe changes in the glycocalyx is essential for elucidating the underlying mechanisms. Currently, numerous techniques for detecting glycocalyx have been reported in the literature, including sidestream darkfield imaging (39), intravital microscopy (40), transmission electron microscopy (37), fluorescent staining (41, 42), and ELISA (43). While intravital microscopy provides direct observations, it poses significant technical challenges. Transmission electron microscopy is technically more operable, but the premise is that it needs to be fixed with a suitable solution and the structure is well preserved to make it a more reliable detection method. Transmission electron microscopy, although more operable, requires fixation with a suitable solution and careful preservation of structure to ensure reliability. Lectins, which are carbohydrate-binding proteins, can bind to fluorescent markers either directly or indirectly and are utilized to identify specific glycoproteins within the endothelial cell surface layer. Although ELISA offers a convenient means for the quantitative detection of glycocalyx components such as HS and HA, it is important to recognize that HS and HA are polysaccharides commonly found on both the cell surface and in the cytoplasmic matrix, and do not specifically represent the endothelial glycocalyx. Using the ELISA method may lead to interference from other factors, potentially skewing experimental results. Therefore, caution is warranted when drawing conclusions based on measurements obtained through this method.

## 5 Factors of glycocalyx degradation

**Figure 2:**
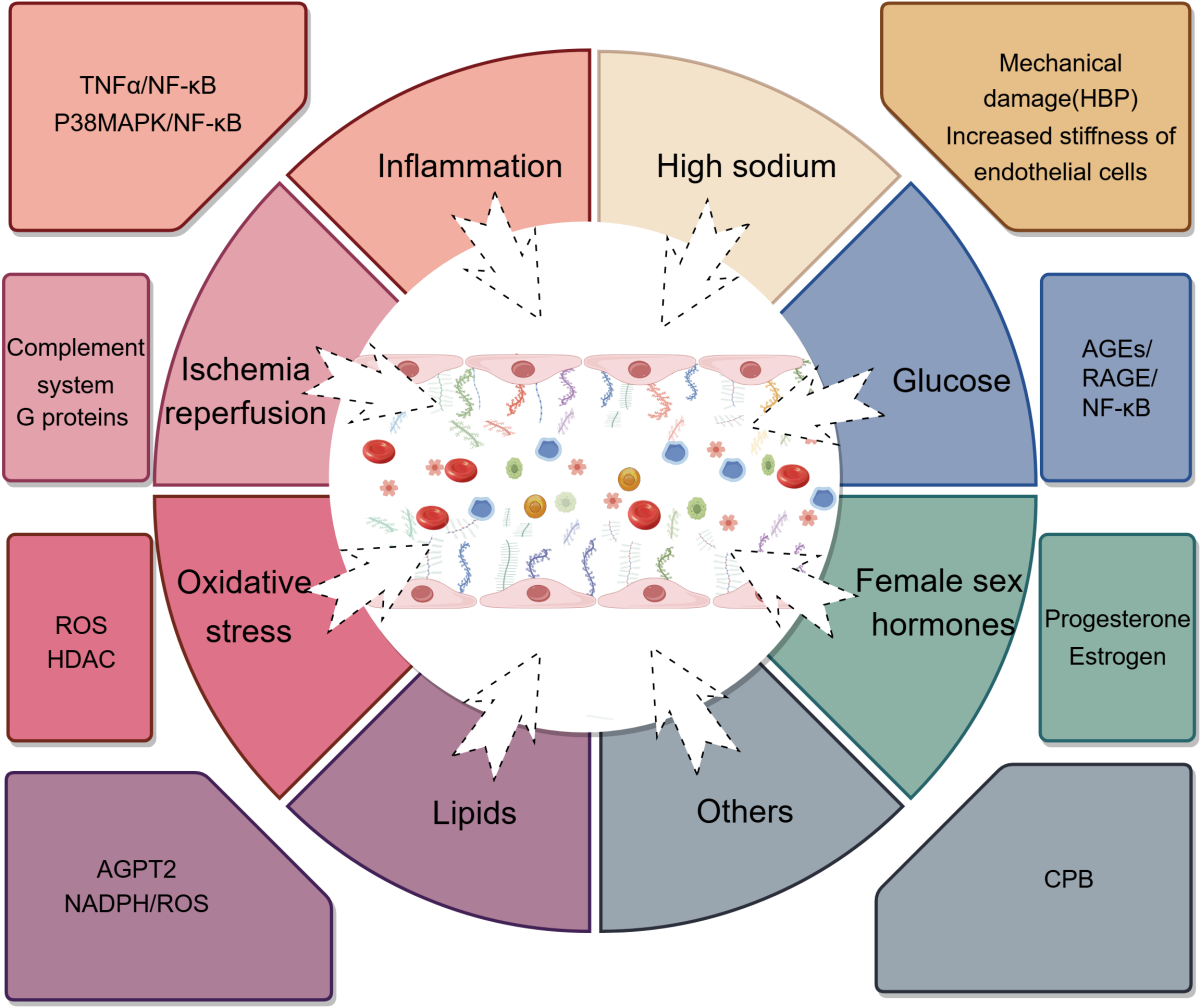
The factors influencing endothelial glycocalyx degradation. TNFα, tumor necrosis factor; NF-κB, nuclear factor kappa-B; P38MAPK, p38 mitogen-activated protein kinase; HBP, high blood pressure; AGEs, advanced glycation end-products; RAGE, receptor of advanced glycation end-products; ROS, reactive oxygen species; HDAC, histone deacetylase; ANGPT2, Angiopoietin-2; NADPH, nicotinamide adenine dinucleotide phosphate; CPB, cardiopulmonary bypass. (By Figdraw).

The reduction in the glycocalyx primarily involves increased degradation and shedding due to the over-expression of glycocalyx-degrading enzymes and inhibited glycocalyx production under the influence of various factors, leading to decreased expression (**Figure 2**). The enzymes responsible for degrading the glycocalyx include matrix metalloproteinases (MMPs), a disintegrin and metalloproteinase (ADAM), heparanase (HPSE), and hyaluronidase (HAase) (8, 44).

### 5.1 Inflammatory factors

Inflammation is the body’s natural defense mechanism against external stimuli. However, prolonged, excessive, and uncontrolled inflammatory responses can cause damage to the body. During inflammation, inflammatory mediators activate and alter endothelial cells, damaging the endothelial barrier function. Glycocalyx, the primary defense line to protect the endothelium, is closely related to endothelial barrier function. Therefore, inflammation has a significant impact on the shedding of the glycocalyx.

Inflammation plays a critical role in the degradation of the glycocalyx. On the one hand, the inflammatory response can promote the upregulation of HPSE1 expression, resulting in the reduction of HS (45). *In vitro* and *in vivo* studies have demonstrated that inflammatory stimuli, including tumor necrosis factor-α (TNF-α) and lipopolysaccharide, can directly induce the upregulation of HPSE expression as well as an increase in HPSE mRNA levels in glomerular endothelial cells and podocytes (46). On the other hand, inflammatory cytokines, such as TNF-α, have been observed in related inflammatory disease models to increase the specific inflammatory sulfation motif of HS, altering its structure. N- and 6-O-sulfated domains in HS on activated glomerular endothelium are crucial for leukocyte trafficking and are possible therapeutic targets (20, 47). N-deacetylase-N-sulfotransferase 1 is a crucial enzyme involved in the modification of HS. *In vitro* experiments demonstrated that silencing the N-deacetylase-N-sulfotransferase 1 gene in mouse glomerular endothelial cells using small interfering RNA led to a reduction in the expression of inflammation-related N- and 6-O sulfated HS domains (48). This alteration may further impact the stability of the glycocalyx, rendering it more susceptible to degradation by HPSE.

Lipowsky (49) mentioned the shedding of the vascular endothelial glycocalyx and the almost simultaneous release of TNF-α in an inflammatory mouse model. Inagawa et al (50) observed a significant decrease in the glycocalyx of pulmonary capillaries during endotoxemia. However, it was unclear whether shedding of the glycocalyx triggered the release of inflammatory mediators or if the release of inflammatory mediators led to the destruction of the glycocalyx. Chappell D et al (43) conducted experiments to elucidate the sequence of inflammatory mediator release and glycocalyx shedding. To measure the HS concentrations, they used an enzyme-linked immunosorbent assay (ELISA) based on two antibodies specific for HS-related epitopes and found that significant shedding of glycocalyx components in coronary vessels has been observed following perfusion of isolated hearts for 20 min with TNF-α (43). This outcome indicated that the activation of TNF-α-related signaling pathways was one of the factors contributing to glycocalyx destruction (51).

Further research revealed that TNF-α activation had a negative impact on glycocalyx integrity through the nuclear factor-kappa B (NF-κB) signaling pathway, resulting in the shedding of the syndecan-4 domain from the surface of endothelial cells in an inflammatory environment (52). The P38MAPK/NF-κB pathway is one of the primary pathways influencing inflammation (53). Silent information regulator 2 - related enzyme 1 (SIRT1) is a NAD+ - dependent deacetylase. During the deacetylation process, SIRT1 removes the acetyl group from substrate proteins, thereby altering their charge state, structure, and function. It has been demonstrated that SIRT1 can inhibit the expression levels of inflammatory genes in various cell types (54), including endothelial cells. The study showed that SIRT1 could bind to the p65 subunit of NF-κB, leading to the deacetylation of Lys310 in p65, resulting in the loss of NF-κB activity and affecting the transmission of the P38MAPK/NF-κB signaling pathway (55). In addition, endothelial cells were deficient in SIRT1 showed activation of the NF-κB pathway and shedding of the external domain of syndecan-4 (56), which suggested that SIRT1 participated in the regulation of P38MAPK/NF-κB pathway and affected the shedding syndecan-4. Numerous plant-derived compounds have been identified as promoters of SIRT1 expression or activity, including resveratrol, quercetin, fisetin, curcumin, berberine, and kaempferol. The efficacy of these natural compounds in specific environments may be linked to potential strategies for preventing glycocalyx damage (57).

Interleukin-1β (IL-1β) is a cytokine primarily produced by mononuclear macrophages and plays a regulatory role in cell-mediated immunity and inflammatory responses. A study found that when stimulated with IL-1β, immortalized glomerular endothelial cells exhibited a 60% reduction in syndecan-4 protein and gene expression and a 50% increase in MMP-9 gene expression (58). Knocking out the MMP-9 gene could reduce syndecan-4 shedding by 50%, and this phenomenon was independent of high glucose conditions (58). Hyperglycemia is also an important factor in promoting MMP-9 expression. The transcription of the MMP-9 gene is regulated by various transcription factors, particularly NF-κB and activator protein-1 (AP-1) (59). The activity of these transcription factors is enhanced under hyperglycemic conditions, suggesting that hyperglycemia may facilitate MMP-9 transcription to some extent. IL-1β promoted the shedding of syndecan-4 in immortalized glomerular endothelial cells by increasing MMP-9 expression in a manner not dependent on high glucose levels. These findings indicate that there are alternative mechanisms through which IL-1β enhances MMP expression and contributes to glycocalyx shedding, warranting further investigation.

Numerous fragments that act as inflammatory mediators are produced during the activation of the complement system. Mutations in the complement factor H gene can lead to its abnormal function. For instance, in atypical hemolytic uremic syndrome, complement factor H fails to perform its regulatory role in the complement system effectively, resulting in over-activation of the complement pathway. This over-activation leads to damage of microvascular endothelial cells and thrombotic microangiopathy, accompanied by inflammation. Inflammatory cells infiltrate the areas surrounding the damaged microvessels, further exacerbating tissue injury. It has been reported that variations in the complement factor H gene influence its interaction with heparan HS, which may, in turn, impact glycocalyx function (60). This observation indirectly suggests that complement factor H may affect the degradation of glycocalyx through mechanisms that remain to be elucidated.

### 5.2 Ischemia reperfusion

Ischemia-reperfusion injury (IRI) refers to tissue damage caused by the restoration of blood flow and tissue oxygenation following a sudden interruption. This phenomenon can occur in various disease processes, such as myocardial infarction, ischemic stroke, and organ transplant surgeries. Research on C57Bl/6 mice models of cardiac IRI observed a thinning of the glycocalyx within five minutes of reperfusion and a decrease in NO-mediated endothelium-dependent vasodilation due to shedding (61). It suggested glycocalyx degradation might be one of the earliest pathological phenomena in IRI. However, there was a study showed that pigs exhibited increased serum HS concentrations after 90 minutes of ischemia, followed by 120 minutes of reperfusion (62). The significant disparity in time between the two research findings warrants further investigation. This difference may be attributed to variations in animal species, experimental environments, or experimental methodologies. Researchers implemented a systemic ischemia model in guinea pigs to determine the origin of syndecan-1 and HS sources during the ischemic phase. They found that compared to non-ischemic guinea pigs, the levels of syndecan-1 and HS in the blood of the systemic ischemia model guinea pigs elevated (63), and the amount of glycocalyx on the aortic vessels reduced, demonstrating that glycocalyx damage was closely associated with ischemia.

Passov, A et al (64) observed early shedding of syndecan-1 and HS in patients undergoing aortic valve replacement surgery. Glycocalyx shedding markers (such as HS and syndecan-1) were also found to elevate after reperfusion in organ transplant surgeries, including lung, liver, and kidney transplants (65–67). It also was confirmed in patients with ischemic stroke, where various soluble glycocalyx components in the plasma, including HS, CS, DS, syndecan-2, and syndecan-3, increased one week after the initial event (68). Furthermore, research revealed that patients undergoing major vascular surgery with global and regional ischemia were also shedding the endothelial glycocalyx, providing further evidence of glycocalyx layer detachment during ischemia (63). However, this did not mean glycocalyx damage only occurs during the ischemic phase. Further research is needed to determine whether glycocalyx damage occurs during ischemia or reperfusion.

MMPs can directly cleave syndecan-1 and HS chains and have been identified as a significant factor in causing glycocalyx shedding under various pathological conditions. Lalu M M et al (69) found that IRI affects the activity of MMPs, thereby speculating that MMPs might be one of the mechanisms by which IRI causes glycocalyx shedding. There was a study that observed the deposition of complement components C3d and C5b-9 in the reperfused hearts of patients with myocardial infarction (70). Given the membrane-attacking nature of the complement system, its activation could contribute to glycocalyx damage during IRI. Conversely, some literature suggested that glycocalyx damage created conditions for activating the complement system (71). Therefore, further exploration is needed to determine the connection between the complement system and glycocalyx degradation in IRI.

A. W. Mulivor mentioned that G proteins could mediate glycocalyx damage during IRI (72). It reported a significant reduction in the composition of the glycocalyx of venular endothelium in postcapillary venules (rat mesentery) under conditions of inflammation and IRI. However, pretreatment with pertussis toxin solution for 30 minutes before reperfusion markedly attenuated the decrease in glycocalyx composition. Pertussis toxin exhibits a strong affinity for G proteins, thereby inhibiting their function. Consequently, these findings suggest that the shedding of the glycocalyx in models of inflammation and IRI may be mediated by lyases released or activated by G protein signaling. It has also been shown that ischemia and reperfusion can result in changes in circulation hemodynamics and cause damage to the glycocalyx. In summary, IRI is a significant factor in damaging the glycocalyx. Currently, glycocalyx degradation is considered the cornerstone of endothelial dysfunction associated with IRI. After glycocalyx damage, additional harm to the local microcirculation occurs to create a vicious cycle of organ damage.

### 5.3 Oxidative stress

Oxidative stress is widely considered a key factor associated with aging (73). Literature indicates that increased age is associated with reduced glycocalyx in aged mice and older people (74), manifested in decreased capillary density and a noticeable reduction in glycocalyx thickness in older individuals (75). In an animal experiment, a reduction in glycocalyx thickness was observed in the brain microvessels of aged mice when examining the ultrastructure of mouse tissues (76). Intermittent fasting has become of interest for its possible metabolic benefits and reduction of oxidative damage, all of which play a role in the pathophysiology of diabetic nephropathy. In a diabetic apolipoprotein E knockout mouse model, the capillary loop morphology and endothelial glycocalyx HS contents were preserved during a simulated fasting diet cycle. These findings suggest that the reduction of oxidative stress is associated with diminished damage to the glycocalyx structure (77).

Reactive oxygen species (ROS) are involved in oxidative stress responses, exacerbating oxidative stress and damaging cellular structures and functions (78). Therefore, ROS produced during inflammatory responses or IRI processes can mediate the collapse of the glycocalyx (79). ROS substances that mediate glycocalyx degradation include hydroxyl radicals, carbonate radical anions, and hypochlorous acid (HOCl). Among these compounds, HOCl can convert GAGs in the glycocalyx into GAG chloramides, making them specific targets for oxidation or reduction, ultimately leading to degradation (80). The antioxidant superoxide dismutase has demonstrated that maintaining glycocalyx integrity is inversely related to ROS in oxidative stress responses.

Studies have shown that the activity of histone deacetylases (HDAC) increases under oxidative stress conditions, leading to the up-regulation of MMP expression (81). It is accompanied by a decrease in the levels of tissue inhibitors of metalloproteinases, ultimately leading to the degradation of the glycocalyx. This suggests that oxidative stress can regulate HDAC activity, influencing MMP expression levels and participating in the degradation process of the endothelial glycocalyx.

### 5.4 Lipids

It has been reported that changes in the concentration and composition of lipoproteins make them the primary factors in glycocalyx damage. The process involves modified low-density lipoproteins (LDL), remnant lipoproteins (Lp), triglyceride-rich lipoprotein (TGRLp) products, and dysfunctional high-density lipoproteins (dysHDL) (82). Researchers used tracers to measure the levels of endothelial glycocalyx in hypercholesterolemic patients and compared them to healthy controls (83). They found that the former had less glycocalyx. However, after treatment with rosuvastatin, an increase in the glycocalyx was observed, suggesting that elevated blood lipids might promote glycocalyx degradation.

A study found a significant correlation between serum syndecan-1 levels in patients with nephrotic syndrome and low-density lipoproteins-cholesterol (LDL-C), high-density lipoproteins-cholesterol (HDL-C), and triglycerides (TG) (84). Researchers observed that nephrotic patients exhibited elevated serum levels of angiopoietin-2 (AGPT2) compared to control subjects. Mediation analysis indicated that AGPT2 may play a role in the relationship between features of nephrotic syndrome, particularly high LDL-C, and the derangement of the endothelial glycocalyx (85). Consequently, the association between LDL-C and glycocalyx degradation in nephrotic syndrome patients could be mediated by AGPT2. AGPT2 is a crucial enzyme in the fatty acid synthesis pathway, primarily responsible for regulating fatty acid synthesis. Changes in its activity and expression levels are closely linked to intracellular lipid metabolism. LDL-C induces a disturbance in intracellular lipid metabolism and increases ROS production by stimulating the expression and activity of AGPT2, which may underlie the mechanism of glycocalyx injury.

Increased perfusion boundary region (PBR) of the sublingual arterial microvessels (ranging from 5 to 9 µm) using Sideview Darkfield imaging was proposed as a valid and non-invasive method to assess endothelial integrity by the European Society of Cardiology Working Group on Peripheral Circulation to measure accurate index of reduced EG thickness. Utilizing the PBR of sublingual microvessels with diameters of 5-9μm (PBR5-9) as a non-invasive indicator to measure glycocalyx thickness, researchers found that elevated HDL-C levels were linked to increased PBR5-9 in male hypertensive patients (86). This result indicated that higher levels of HDL-C serve as a protective factor for the glycocalyx. Further study revealed that the protective effect of HDL-C on the glycocalyx was more pronounced in the 71-101mg/dL range (87).

Lectin-like oxidized low-density lipoproteins receptor-1 (LOX-1) is a 50 kDa transmembrane glycoprotein that belongs to the C-type lectin family and has a short cytoplasmic tail region, a transmembrane region, and an extracellular carbohydrate recognition domain (CRD). The CRD domain is the key structural basis for LOX-1 to recognize oxidized low-density lipoproteins (ox-LDL). The interaction between LOX-1 and ox-LDL can induce the expression of NADPH oxidase, leading to excessive production of ROS, which causes damage to the glycocalyx and stimulates apoptosis (88). However, antioxidant enzymes, such as superoxide dismutase and catalase, can neutralize this effect (89). Additionally, studies have proven that TNF-α can upregulate the expression of LOX-1 in endothelial cells, enhancing ox-LDL uptake (90). Hence, while glycocalyx degradation is relevant to lipid overstimulation, the degradation associated with ox-LDL may be influenced by inflammation.

### 5.5 Glucose

Glycocalyx mimetic supplementation (EndocalyxTM) can maintain microvascular endothelial health in diabetic patients, indirectly suggesting a potential link between glycocalyces and blood glucose levels (91). Studies have shown that a high glucose environment can significantly reduce the thickness of the glycocalyx, especially in patients with microalbuminuria (92). Researchers speculated that its mechanism might be mediated by oxygen free radicals or the activation of glycocalyx-degrading enzymes, leading to microalbuminuria reflecting glycocalyx damage. Previous findings indicate that hyperglycemic conditions induce mitochondrial fragmentation, which is causal for ROS overproduction (93). Subsequently, Tianzheng Yu proposed that the increased production of ROS from mitochondria is a primary cause of hyperglycemic complications (94). Additionally, cytoplasmic ROS has been reported to be significantly influenced by hyperglycemic states (95). As previously noted, ROS is a major contributor to glycocalyx impairment. This implies that the mechanism by which hyperglycemia leads to glycocalyx damage may be mediated by ROS.

Furthermore, Crompton M and colleagues discovered that exposing human glomerular endothelial cells to high glucose increased MMPs and caused damage to the glycocalyx (96). Another study observed that endothelial damage under high glucose conditions was closely related to increased HPSE levels (97), an enzyme that degrades the glycocalyx. In the investigation of the molecular mechanisms underlying HPSE-induced production in diabetic nephropathy, the researchers identified the transcription factor early growth response protein 1 as a key activator of the HPSE promoter in the context of diabetes (98). In patients with diabetes, there is an increase in HAase activity, which may decrease both glycocalyx volume and functional capillary density (39). In addition, it was found that the loss of HA synthesis in endothelial cells of diabetic nephropathy mice can promote the degradation of glycocalyces, and the mechanism is related to the inactivation of hyaluronic acid synthase 2 gene. These findings validated that hyperglycemia might degrade the glycocalyx by affecting glycocalyx-degrading enzymes.

Long-term hyperglycemia can elevate the production of advanced glycation end-products (AGEs), activating various intracellular signals through receptor and non-receptor-mediated mechanisms. For example, AGEs can activate the receptor for advanced glycation end-products (RAGE) on endothelial cells, leading to their shedding and the formation of soluble RAGE. The soluble form of RAGE can activate the NF-κB pathway, triggering an inflammatory cascade and leading to glycocalyx shedding (99). In summary, the rise in glucose production results in the creation of AGEs and further damage to the glycocalyx. The formation of RAGE elucidates the connection between the glycocalyx and AGEs.

Zuurbier (100) observed the permeability of the endothelial glycocalyx to dextrans in acute hyperglycemia C57BL/6 mice and then discovered that it increased, indicating that vascular permeability caused by hyperglycemia results from increased glycocalyx permeability. Nieuwdorp infused healthy volunteers with 15 mmol/L of glucose for 6 hours and observed a 50% reduction in glycocalyx volume and an increase in plasma HA levels compared to the control group (101). The infusion of N-acetylcysteine could improve this condition. These data suggested that damage to the glycocalyx damage potentially contributes to endothelial dysfunction mediated by hyperglycemia.

### 5.6 High sodium

A cross-sectional study found that newly diagnosed, untreated hypertensive patients had a significantly lower glycocalyx thickness compared to healthy individuals (102). Additionally, those with reduced glycocalyx thickness exhibited signs of significant vascular dysfunction. However, it remains to be further investigated whether the reduction in glycocalyx in hypertensive patients is primarily due to mechanical damage caused by increased blood pressure or high sodium intake. Xiangyu Zheng (103) discovered that a high-salt diet diminished the barrier function of the microcirculation and glycocalyx. Further research revealed that with every 2% increase in plasma sodium concentration above 140 mmol/L, the stiffness of endothelial cells increased by approximately 20% (104). Another related study demonstrated that five days of sodium overload destabilized approximately 50% of the glycocalyx and resulted in a 68% shedding of HS (105).

### 5.7 Female sex hormones

Estrogen and progesterone are significant hormones in women, essential for the reproductive and fetal development and physiological functions of the reproductive system. They also have multiple effects on other organs, including the vascular endothelium. A study examining glycocalyx molecule levels in the serum of healthy women and men found that in healthy women, the levels of glycocalyx molecules in the blood varied with the ovulation cycle. Specifically, the concentrations of HS and Syndecan-1 peaked during the luteal phase, suggesting that products of the luteal phase may be involved in glycocalyx degradation (106). To elucidate the mechanism of glycocalyx degradation, researchers conducted further observations and found that progesterone was the primary substance affecting serum glycocalyx molecule levels (107). Additionally, a study found that patients undergoing controlled ovarian stimulation showed increased immune system activity, as indicated by elevated levels of CRP, TNF-α, and leukocytes (108–110). The increase in TNF-α levels was positively associated with progesterone (111). Thus, the impact of progesterone on the glycocalyx might be mediated by inflammation. However, other studies showed that estrogen could repair the glycocalyx after traumatic shock and promote its barrier-protective function (112).

### 5.8 Others

The cause of glycocalyx degradation is due to a microcirculatory disorder during cardiopulmonary bypass (CPB) (113). A study found that the concentrations of MMP-2 and MMP-9 in patients’ serum significantly increased after CPB, indicating that CPB-induced synthesis and release of MMP-2 and MMP-9 might be the cause of glycocalyx damage (114, 115). However, further research is needed to explore more detailed mechanisms.

## 6 Conclusions

The destruction of the glycocalyx is a primary factor in endothelial dysfunction and subsequent vascular injury. Glycocalyx damage is closely associated with the development of various chronic diseases. Chronic damage to the glycocalyx can result in increased vascular permeability, stiffening of the vessel walls, platelet aggregation, and developing pro-inflammatory and pro-thrombotic conditions. These pathological changes play a significant role in developing various diseases (116). Therefore, identifying the factors and mechanisms impacting glycocalyx damage is crucial for preventing and treating diseases by targeting glycocalyx.

## 7 Outlook

Although previous researchers have acknowledged the significance of glycocalyx in disease development, more in-depth, specific and detailed research is still needed. To enhance the application of the glycocalyx in diagnosing, monitoring, preventing and treating diseases, it is essential to develop more sensitive, accurate, and convenient technologies for detecting glycocalyx-related indicators. This will facilitate early diagnosis of disease, assessment of severity and prognosis. Additionally, a deeper understanding of the specific changes in the glycocalyx across different diseases and how these alterations influence disease progression is necessary. The data have shown a potentially protective effect of GAG in experimental glomerulonephritis and demonstrated that exogenous HSglx reduces albuminuria during glomerulonephritis (117). Such measures could bolster the potential of glycocalyx as a biomarker for multiple diseases and guide the development of new therapeutic strategies and drugs.
